# Cruciferous vegetables as a treasure of functional foods bioactive compounds: Targeting p53 family in gastrointestinal tract and associated cancers

**DOI:** 10.3389/fnut.2022.951935

**Published:** 2022-08-04

**Authors:** Saikat Mitra, Talha Bin Emran, Deepak Chandran, B. M. Redwan Matin Zidan, Rajib Das, Sukamto S. Mamada, Ayu Masyita, Mirnawati Salampe, Firzan Nainu, Mayeen Uddin Khandaker, Abubakr M. Idris, Jesus Simal-Gandara

**Affiliations:** ^1^Department of Pharmacy, Faculty of Pharmacy, University of Dhaka, Dhaka, Bangladesh; ^2^Department of Pharmacy, BGC Trust University Bangladesh, Chittagong, Bangladesh; ^3^Department of Pharmacy, Faculty of Allied Health Sciences, Daffodil International University, Dhaka, Bangladesh; ^4^Department of Veterinary Sciences and Animal Husbandry, Amrita School of Agricultural Sciences, Amrita Vishwa Vidyapeetham University, Coimbatore, Tamil Nadu, India; ^5^Faculty of Pharmacy, Hasanuddin University, Makassar, Indonesia; ^6^Sekolah Tinggi Ilmu Farmasi Makassar, Makassar, Indonesia; ^7^Centre for Applied Physics and Radiation Technologies, School of Engineering and Technology, Sunway University, Subang Jaya, Selangor, Malaysia; ^8^Department of Chemistry, College of Science, King Khalid University, Abha, Saudi Arabia; ^9^Research Center for Advanced Materials Science (RCAMS), King Khalid University, Abha, Saudi Arabia; ^10^Nutrition and Bromatology Group, Department of Analytical Chemistry and Food Science, Faculty of Science, Universidade de Vigo, Ourense, Spain

**Keywords:** cruciferous vegetables, foods bioactive compounds, gastrointestinal cancer, p53 family, apoptosis

## Abstract

In the past few years, phytochemicals from natural products have gotten the boundless praise in treating cancer. The promising role of cruciferous vegetables and active components contained in these vegetables, such as isothiocyanates, indole-3-carbinol, and isothiocyanates, has been widely researched in experimental *in vitro* and *in vivo* carcinogenesis models. The chemopreventive agents produced from the cruciferous vegetables were recurrently proven to affect carcinogenesis throughout the onset and developmental phases of cancer formation. Likewise, findings from clinical investigations and epidemiological research supported this statement. The anticancer activities of these functional foods bioactive compounds are closely related to their ability to upregulate p53 and its related target genes, e.g., p21. As the “guardian of the genome,” the p53 family (p53, p63, and p73) plays a pivotal role in preventing the cancer progression associated with DNA damage. This review discusses the functional foods bioactive compounds derived from several cruciferous vegetables and their use in altering the tumor-suppressive effect of p53 proteins. The association between the mutation of p53 and the incidence of gastrointestinal malignancies (gastric, small intestine, colon, liver, and pancreatic cancers) is also discussed. This review contains crucial information about the use of cruciferous vegetables in the treatment of gastrointestinal tract malignancies.

## Introduction

Our cells are always exposed to a wide array of cellular stresses, such as ionizing radiation, oncogenes, and oxidative stress. Following the exposure of these stresses, genomic aberration and instability may occur which could lead to cancer development. Therefore, a proper and delicate mechanism is required for detecting any DNA damage and protecting cells from malignancies. It has been known that the role of the p53 family (p53, p63, and p73 proteins) in suppressing the development of cancerous cells is indispensable. More than 50% of human cancers are closely linked to the mutation or deletion of p53 which indicates the pivotal role of this transcription factor (1, 2). Once cellular stressors, such as ionizing radiation, are exposed to cells, p53 is activated. This activation could lead to cell cycle arrest and apoptosis. While the former is aimed to repair the damage occurring in DNA due to the stressors exposure, the latter is activated if the damage cannot be repaired.

Of several cancers, gastrointestinal-related cancers, such as colorectal cancer, have been attracting big interest as their malignancies are associated with a high figure of death. In the United States, colorectal cancer-related death is placed in second place. From ~148,000 cases of colorectal cancer in the United States in 2020, the death was estimated at the rate of almost 35% (3). Surprisingly, from this figure, ~18,000 cases were detected in individuals under 50 years old (3).

The efforts conducted to discover and develop novel anticancer candidates from various sources, including from cruciferous vegetables, are still ongoing. These vegetables are a member of Brassicaceae (Cruciferae). It has been known that consumption of cruciferous vegetables, such as broccoli and cauliflower, is correlated with the lower incidence of chronic diseases, including cancer (4, 5). In addition to their nutritive contents (e.g., proteins, carbohydrates, vitamins, and minerals) (6), these vegetables also contain a number of metabolites that can promote health e.g., glucosinolates, isothiocyanates, methyl cysteine sulfoxide, terpenes, anthocyanins, and flavonoids (7–10). Some studies have reported the anticarcinogenic activity of several metabolites found in cruciferous vegetables. For example, glucosinolates, a secondary metabolite mainly found in cruciferous plants, display activity in preventing the development and progression of gastrointestinal cancers as well as in treating these cancers (11). To date, the existence of more than 130 glucosinolates has been reported which can be classified into three major structural groups i.e., aliphatic, indole, and aromatic glucosinolates (12). Biologically, the parent glucosinolate possesses negligible activity in preventing cancer progression. However, once it is converted into its derivatives, such as isothiocyanates, thiocyanates, nitriles, and indoles, the biological activity becomes potent. This conversion is mediated by the action of myrosinases during the mechanical digestion (e.g., chewing and cutting) of the vegetables (13). The anticancer activity of the cruciferous metabolites might be associated with several proposed mechanisms of action. It has been suggested that isothiocyanates and indoles play a pivotal role in regulating cell growth and cell cycle arrest which is essential in repairing the damaged DNA so that this prevents DNA alteration. The pro-apoptotic activity of these metabolites has also been reported. Other putative mechanisms include their activity in modulating oxidative stress and preventing the occurrence of angiogenesis (7). Anticancer activities of these metabolites are closely linked to their ability in upregulating p53 and its related target genes, e.g., p21 (14–16).

This review summarizes the metabolites obtained from various cruciferous vegetables and their potential uses in modifying the tumor-suppressive action of p53 proteins. Further, the correlation between the incidence of gastrointestinal cancers (gastric, small intestine, colon, liver, and pancreatic cancers) and the mutation of p53 is also described. This review provides information that is important for those who are interested in investigating further utilization of cruciferous vegetables in tackling cancers developed in the gastrointestinal system.

## Functional bioactive compounds from cruciferous vegetables

Cruciferous vegetables, often called Brassicaceae or mustard family, are commonly consumed globally in the human diet (17, 18). Brassica genus is the most popular among the Brassicaceae family and consists of 37 different species (19). *Brassica oleracea* are the principal Brassica vegetable species including cabbages, broccoli, brussels sprouts, kale, cauliflower, and others. Their cultivars are classified into seven main groups based on morphology and developmental forms: *B. oleracea* “capitata group” (cabbages), alboglabra group (Chinese broccoli or Kai-lan), acephala group (kale and collard greens), botrytis group (broccoflower, cauliflower, and Romanesco broccoli,), italica group (broccoli), gongylodes group (kohlrabi), and gemmifera group (Brussels sprouts) (17). *Brassica rapa* includes turnip, Asian greens, bok choy, Japanese mustard spinach, mizuna, rapini and napa cabbage are also consumed (Nikolov, 2019). Other species of Brassica are *Brassica hirta* (white mustard), *Nasturtium officinale* (watercress), *Amoracia rusticana* (horseradish), *Eruca vesicaria* (arugula), *Lepidium sativum* (garden cress), *Raphanus sativus* (radish), and *Wasabia japonica* (wasabi) (17). Cruciferous vegetables are high in nutrients and secondary metabolites such as sulfur-containing compounds, phenolic compounds, carotenoids, and others, known to have beneficial health properties.

### Nutrients

Cruciferous vegetables have high macronutrients and micronutrients (20). Macronutrients are the nutritive components that provide energy and are required to maintain body functions. The macronutrients of different Brassica vegetables is shown in Table 1. Water is the main component of these vegetables, with ranges 89–92%, while the fiber and fat content are relatively low (6, 8). Brussels sprouts possess high carbohydrates compared to other vegetables (21).

**Table 1:** Macronutrients of cruciferous vegetables per 100 g.

**Species**	**Cultivar**	**Common** **name**	**Energy** **(kcal)**	**Water** **(g)**	**Carbohydrates** **(g)**	**Proteins** **(g)**	**Fats** **(g)**	**Fiber** **(g)**
*Brassica oleracea*	var. *italica*	Broccoli	34	89.30	6.64	2.82	0.37	2.60
	var. *gemmifera*	Brussels sprouts	43	86	8.95	3.38	0.30	3.80
	var. *capitata*	Cabbage	25	92.18	5.80	1.28	0.10	2.50
	var. *botrytis*	Cauliflower	25	92.07	4.97	1.92	0.28	2.00
	var. *viridis*	Collard greens	32	89.62	5.42	3.02	0.61	4.00
	var. *achepala*	Kale	35	89.63	4.42	2.92	1.49	4.10
*Brassica juncea*	var. *rugosa* (or *integrifolia*)	Mustard greens	27	90.70	4.67	2.86	0.42	3.20
*Brassica rapa*	ssp. *rapa*	Turnip	28	91.87	6.43	0.90	0.10	1.80
	ssp. *parachinensis*	Rapini, broccoli rabe	22	92.55	2.85	3.17	0.49	2.70
*Raphanus sativus*	-	Radish	16	95.30	3.40	0.70	0.10	1.60

Moreover, cruciferous vegetables are also rich of micronutrients (Table 2) such as minerals (calcium, potassium, magnesium, iron, phosphorus, sulfur, chlorine, sodium, zinc, and selenium) and vitamins (thiamine, riboflavin, niacin, folate, and tocopherol) (22). These vegetables contain calcium in the range of 22–150 mg/100 g. Kale is an important mineral source especially potassium, magnesium, calcium, and iron. On the other hand, kale also contains a high level of vitamin C and folates (19, 23). Broccoli and brussels sprouts comprise potentially useful amounts of nutritionally important minerals. In a fresh state, these vegetables also consist of catalase, peroxidase, and superoxide dismutase enzymes. However, the nutritional constituent of Brassica vegetables depends on diversity, growth environment, time of harvest, processing, and cooking conditions (11).

**Table 2:** Micronutrients of cruciferous vegetables per 100 g.

**Cruciferous plant**	**Edible part**	**Minerals (mg)**				**Vitamins**	
		**K**	**Ca**	**Mg**	**Fe**	**C (mg)**	**B9 (μg)**
Broccoli	Inflorescence	316	47	21	0.7	89.2	63
Brussels sprouts	Buds	389	42	23	1.4	85	61
Cabbage	Leaves	170	40	12	0.5	36.6	43
Cauliflower	Inflorescence	299	22	15	0.4	48.2	57
Collard greens	Leaves	-	232	27	-	35.30	-
Kale	Leaves	491	150	47	1.5	120	141
Mustard greens	Leaves	384	115	32	1.64	70	12
Turnip	Root	191	30	11	0.3	21	15
Rapini, broccoli rabe	Leave, stem, flower buds	-	108	0.39	-	20.20	-
Radish	Root	233	25	10	0.3	14.8	25

### Sulfur-containing compounds

**Figure 1:**
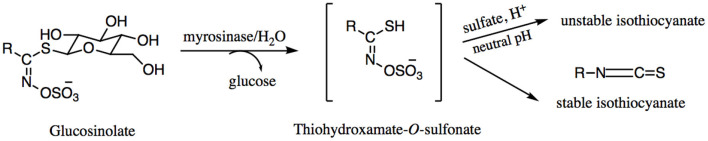
Enzymatic hydrolysis pathways of glucosinolate.

Among the bioactive compounds in brassica vegetables, sulfur-containing compounds such as glucosinolates are the major constituents (24). Glucosinolates are found in these vegetables in high quantities of 1,500–2,000 μg/g, especially in broccoli, brussels sprouts, and cabbage (19). Glucosinolates are responsible for their spicy taste and pungent odor (25). These compounds are water-soluble anions with the basic structure consisting of a β-D-glucopyranose moiety and β-thioglucoside N-hydroxysulfate with a variable side chain derived from amino acids (26). The glucosinolates are biosynthesized in brassica plants by major three steps of naturally occurring chemical reactions namely, side chain modification, side chain elongation and glucone biosynthesis (8). A small change in the side chain determines its classification, such as aliphatic (e.g., glucobrassicanapin, glucoalyssin, glucoraphanin, glucocapparin, dehydroerucin, glucoerucin, epi-progoitrin, glucoiberin, glucoerysolin, glucolepidin, progoitrin, and sinigrin), aromatic aryl (e.g., glucobarberin, glucotropaeolin, gluconasturtiin, glucosinalbin, and glucosibarin), and aromatic indoles (neoglucobrassicin, glucobrassicin, 4-methoxyglucobrassicin, and 4-hydroxyglucobrassicin) (27, 28). The hydrolysis pathways of glucosinolate can be seen in Figure 1.

**Figure 2:**
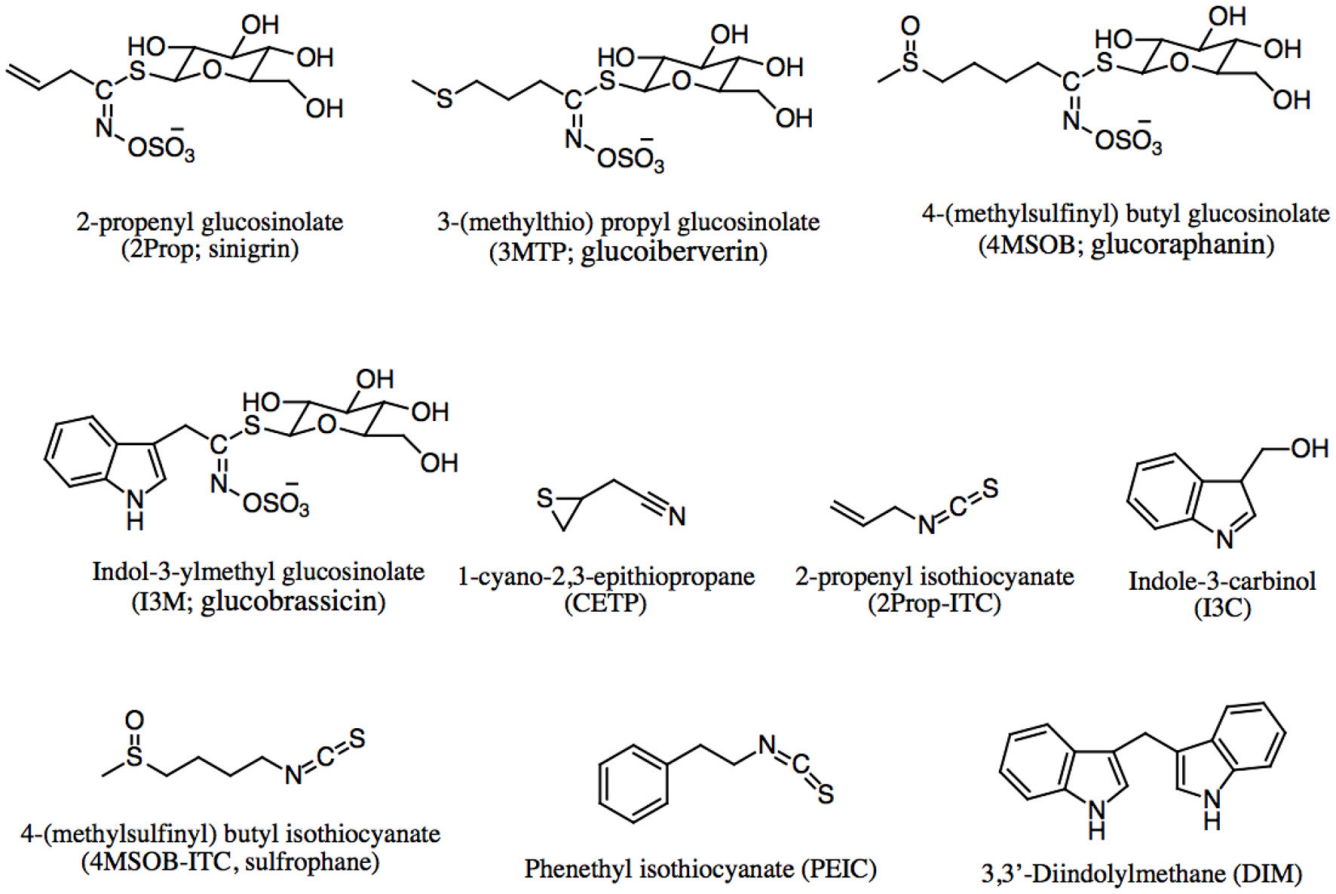
Chemical structures of glucosinolate and their breakdown products.

Glucosinolates are chemically and thermally stable, but not biologically active until they are hydrolyzed (Figure 1) by the β-thioglucosidase or myrosinase enzymes. This enzyme is released after damage in the plant by chewing or processing such as cutting, chopping, and mixing (19). Upon the plant cells are injured, the thioglucosidic bond breaks down, β-thioglucoside yields a β-D-glucose molecule and thiohydroximate-O-sulfonate (an unstable aglycone) (29). Then, some breakdown products are formed depending on the pH level and other conditions (30). Hydrolysis of glucosinolates can also occur by gut microbiota action (25, 31). Both glucosinolates and their breakdown products play a large role in the health benefits of cruciferous vegetables. The more common breakdown products (Figure 1) include isothiocyanates (ITC), thiocyanate, amines, epithionitriles, indolic alcohols, nitriles, and oxazolidinethions (Figure 2) (12). The type and concentration of glucosinolates depends on cultivar, cultivation site, genotype, growth conditions, storage conditions, plant stage, preparation and cooking techniques (32). For example in cooking methods, glucosinolate levels were maximized in steaming compared to microwaving, boiling, and pressure cooking (33). Moreover, Casajús et al. found that the content of aliphatic glucosinolates decreased after storing broccoli in the darkness (34).

In a study by Hanschen et al., the formation of glucosinolates and their hydrolysis products of the five *Brassica oleracea* varieties including broccoli, cauliflower, and white, red, as well as savoy cabbages were determined. They reported that broccoli and red cabbage contains highest level of 4-(methylsulfinyl) butyl glucosinolate (glucoraphanin), whereas cauliflower, white cabbage and savoy cabbage mainly rich in 2-propenyl (sinigrin) and 3-(methylsulfinyl) propyl glucosinolate (glucoiberin). The sprouts of white cabbage is a rich source of the hydrolysis products such as epithionitriles or nitriles with 1-cyano-2,3-epithio propane with up to 5.7 μmol/g fresh weight. They also figured out that mini vegetable heads contained the highest concentrations of isothiocyanate (35). Similarly, Bhandari et al. evaluated the profiles of nine Brassica crops (baemuchae, broccoli, cabbage, Chinese cabbage, cauliflower, kale, leaf mustard, pakchoi, and radish) in various tissues: seeds, sprouts, mature roots, and shoots. Their results showed that total glucosinolate levels in most Brassica crops were highest in seeds and lowest in shoots. Aliphatic, indole, and aromatic glucosinolates were highest in the seeds, shoots, and roots tissues in most of the crops, respectively. The highest total glucosinolate levels were observed in seed and sprout of broccoli (110.76 and 162.19 μmol/g), whereas radish exhibited the lowest total glucosinolate levels across all tissues examined. On the other hand, leaf mustard showed the highest total concentration of glucosinolate in shoots (61.76 μmol/g) and roots (73.61 μmol/g) (36).

### Carotenoids and tocopherols

**Figure 3:**
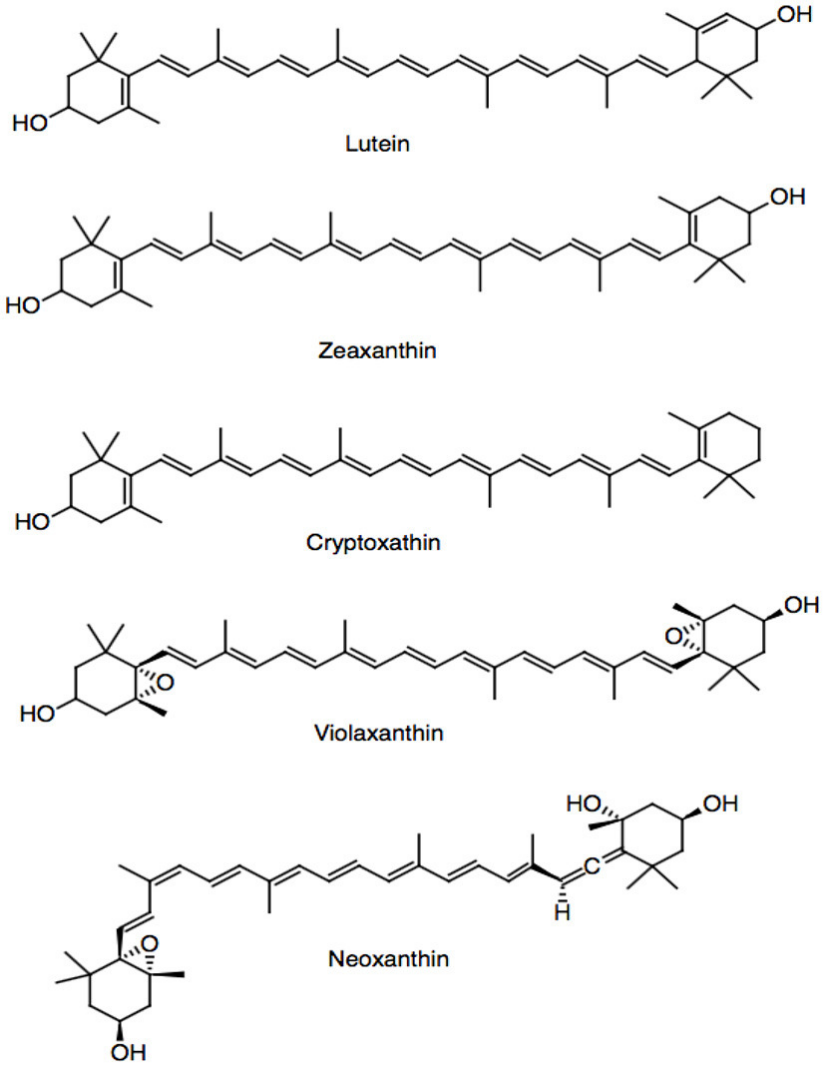
Carotenoids present in cruciferous vegetables.

Carotenoids are highly pigmented constituents (red, yellow, or orange) and also responsible for the appearance of the cruciferous vegetables (37, 38). These phytochemicals are classified as symmetrical tetraterpenes that possess a C40 backbone structure with conjugated double bonds. A wide range of carotenoids are observed in these vegetables including lutein (Figure 3) and β-carotene as the predominant ones, followed by α-carotene, γ-carotene, β-cryptoxanthin, and lycopene presenting an antioxidant activity (39). Other carotenoids such as zeaxanthin, cryptoxanthin, neoxanthin, and violaxanthin have also been found in variable amounts (21, 40, 41).

Among the cruciferous vegetables, kale is considered the richest source of carotenoids, exceeding cabbage in about 40 times (18, 42). The main carotenoids present in kale are zeaxanthin and lutein, but important differences were identified among several cultivars of kale (41, 43, 44). Similarly to glucosinolates, the carotenoid accumulations in the brassica family are regulated by the developmental stage, environment, and tissue type (45). Tocopherols and tocotrienols (vitamin E) are lipid-soluble compounds present in cruciferous vegetables (39). Regarding the main tocopherols, α-tocopherol has been reported, but γ-tocopherols and δ-tocopherols are also detected (46, 47).

### Phenolic compounds

**Figure 4:**
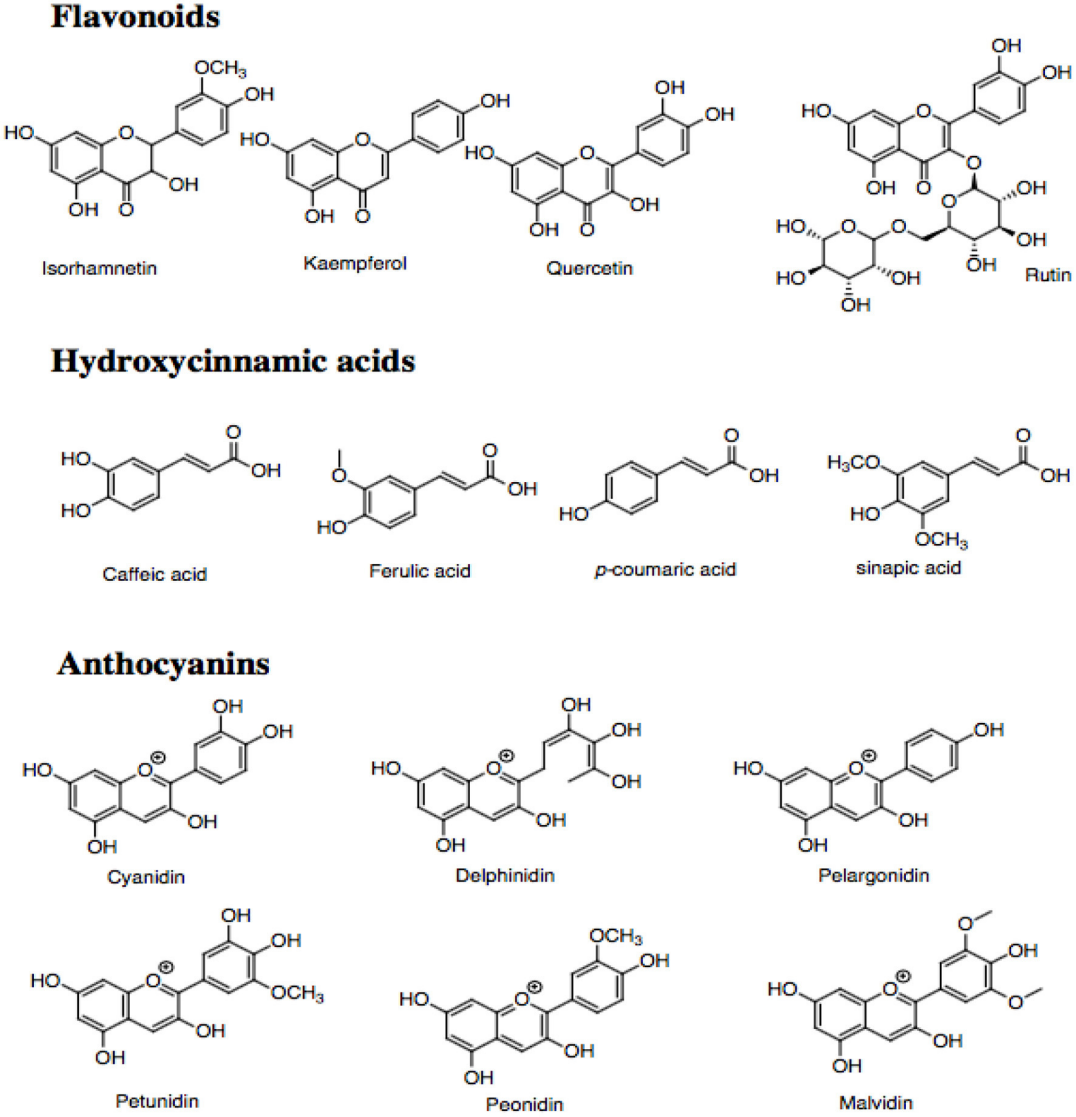
Phenolic compounds present in cruciferous vegetables.

Phenolic compounds are ubiquitously distributed bioactive compounds found in most plant tissues, including cruciferous vegetables (10, 48). These compounds are characterized by having at least one aromatic ring and one or more hydroxyl groups bonded directly to the former, showing diversity of structures (49). Their categorized based on the number and arrangement of carbon atoms in flavonoids (flavones, flavonols, flavanones, flavan-3-ols, isoflavones, and anthocyanidins) and non-flavonoids (phenolic acids, hydroxycinnamates, and stilbenes) (48, 50–52). Cruciferous vegetables possess antioxidant activity attributes to the high contents of phenolic compounds (53, 54). The most important phenolic constituents (Figure 4) present in cruciferous vegetables are the hydroxycinnamic acids and the flavonoids such as kaempferol, isorhamnetin, quercetin, and rutin (11, 18).

The profile of phenolic compounds vary significantly among species. In studies carried out by Upadhyay et al., red cabbage presented high contents of rutin (102.14 μg/g of fresh weight) compared to green cabbage (1.34 μg/g of fresh weight) and cauliflower (39.07 μg/g of fresh weight). The sinapic acid content of red cabbage, broccoli, cauliflower, and green cabbage are 30.20, 7.37, 4.66, and 3.52 μg/g of fresh weight, respectively. They also detected that broccoli had a high content of gentisic acid (83.17 μg/g of fresh weight), followed by cauliflower and green cabbage with values of 35.50 and 2.50 μg/g of fresh weight, respectively (55). Furthermore, Li et al. identified 74 phenolic compounds of 12 different cruciferous vegetables, including 58 flavonoids and 16 hydroxycinnamic acids and their derivatives using ultra-high performance liquid chromatography-quadrupole time-of-flight mass spectrometry (UHPLC-Q-TOF-MS/MS). Among the flavonoids, the main compounds found were kaempferol, isorhamnetin, and glycosylated quercetin. The main hydroxycinnamic acids were caffeic, ferulic, sinapic, and *p*-coumaric acids. Emphasizing that the cauliflower and cabbage presented high contents of flavonoids (5.70 mg/g dry weight) and hydroxycinnamic acids (46.02 mg/g of dry weight) (48, 56, 57).

Another group of phenolic compounds frequently detected in Brassicaceae vegetables is anthocyanins (18). They are responsible for pigmentations of red cabbage and purple cauliflower. The common anthocyanins in Brassica crops are cyanidin, delphinidin, pelargonidin, petunidin, peonidin, and malvidin (21, 58). Broccoli sprouts and red cabbage contain mainly cyanidin glucosides derivatives (59). Red radish contains mainly cyanidin and peonidin anthocyanins acylated with aromatic acids (60).

### Others

Phytosterols are other compounds found in cruciferous vegetables (21). They are steroid alcohols with a molecular nucleus of 17 carbon atoms and a characteristic three-dimensional arrangement of four rings. *Brassica napus* L., known as rapeseed, is the rich source of phytosterols among cruciferous vegetables, with yields of up to 9.79 g/kg oil (18, 61). In addition, *Brassica juncea* is also the most abundant natural source of phytosterols (62).

Brassicaceae family containing phytoalexins possess an indolic ring with C3 substitutions with N and S atoms (63, 64). Phytoalexins are produced from brassinin, which confers a unique structure among other vegetables (64). They have been identified in *Brassica juncea, Brassica napus, Brassica oleracea, Sinapis alba, Raphanus sativus, and Wasabi japonica* (18, 65). Furthermore, fatty acids such as palmitic, stearic, oleic, linoleic, linolenic, eicosenoic, erucic, arachidic, arachidonic, and behenic acid also present in the oil extracted from Brassica crops (66).

## Pathophysiology of p53 family in gastrointestinal cancers: Current status

As the “guardian of the genome,” the p53 family (p53, p63, and p73) plays a critical role in preventing the development of cancers associated with DNA damage (67). Following the activation of the p53 family by a number of cellular stress (e.g., oncogenes, oxygen deficiency in the blood, radiation, and oxidative stress), several mechanisms are activated as the mitigating system toward the affected DNA i.e., cell cycle arrest, apoptosis, and senescence (68). Given the crucial role of p53 in suppressing cancer development, mutations causing dysfunctionality of p53 put the body in danger. Mutations occurring in the p53 family have been found in more than half of human cancers (1). Of this figure, p53 family-related mutations are detected in more than 50% of gastrointestinal cancer cases (69). It has been demonstrated that most p53 mutations are identified as missense mutations with DNA-binding domain (DBD) as the main site for the mutations (67).

The three members of the p53 family are encoded by p53, p63, and p73 genes located in different chromosomes i.e., 17p13, 3q27-29, and 1p36, respectively (69). Nevertheless, they share the high identity of amino acid sequence in the main three structural domains namely transactivation (TAD), DBD, and oligomerization domains (OD) (68). The former domain is responsible for regulating the transcription activity of p53 by providing the binding sites for either positive or negative regulators of p53. Further, as the central domain, the DBD is important for binding to response elements of various target genes. Finally, the latter domain acts as the main site for DNA alternative splicing and post-translational modification. In the normal physiological condition, the expression of p53 in the cell is very low. Upon the exposure of the aforementioned cellular stress to the cells, p53 upregulates the expression of its main negative regulator namely murine/human double minute 2 (MDM2) which provides a negative feedback mechanism to maintain the minute levels of p53 in normal cells (68).

### p53 and apoptosis

As stated above, activation of p53 could induce apoptosis through either the intrinsic or extrinsic pathways. While the former pathway involves the role of mitochondria, the latter apoptotic pathway is induced by death receptors (DRs) (70). Molecularly, the intrinsic pathway of apoptosis is initiated by the upregulation of several B-cell lymphoma-2 (Bcl-2) pro-apoptotic proteins (e.g., Bax, Bak, Noxa, and PUMA) and downregulation of Bcl-2 pro-survival proteins (e.g., Bcl-2, Bcl-w, and Bcl-XL) (71). Upon cellular stress, Bax and Bak experience oligomerization leading to the release of cytochrome c from the intermembrane space of mitochondria to the cytosol (72). As a response to the release of cytochrome c, oligomerization of the apoptotic protease activating factor-1 (APAF-1) occurs followed by the formation of apoptosome complex. This complex recruits and activates pro-caspase-9, an initiator caspase of the intrinsic apoptotic pathway, which is subsequently followed by the induction of several executioner caspases, mainly caspase-3 (73).

The extrinsic pathway of apoptosis involves the role of the superfamily of tumor necrosis factor receptors (TNFRs), also known as DRs, such as TNFR1, DR3, DR6, CD4 (TRAIL-R1), CD5 (TRAIL-R2), and CD95 (67, 74, 75). These receptors are characterized by the presence of the “death domain” in their structure located in the cytoplasmic region. Upon binding of the appropriate ligand to the DRs, several adaptor proteins are activated followed by the recruitment of the caspase-8 and caspase-10 known as the initiator caspases. Eventually, this cascade activates caspase-3 resulting in the occurrence of apoptotic events (74).

### p53 and cell cycle arrest

Another mechanism exerted by p53 as a response to cellular stress is the activation of cell cycle arrest. This mechanism acts as the checkpoint in which the cell is evaluated, checked, and repaired if there is damage, before moving to the other phases in the cell cycle. It has been known that the cell cycle is delicately regulated by cyclins. These proteins exert their actions on controlling the cell cycle through their interaction with another protein belonging to the cyclin-dependent kinases (CDKs) family (68). The activation of these proteins is essential for driving the cell cycle. Conversely, the inhibition of the CDKs could stop the cell cycle so that no cellular duplication and division occurs which is important in blocking the progression of cancer cells. At this point, the involvement of p21 is crucial as this protein could inhibit the CDKs. It has been demonstrated that p53 could induce the activation of p21 following cellular stress exposure. In addition to p21, other target genes of p53 are involved in facilitating cell cycle arrest. Some of those genes are 14-3-3σ, GADD45, and retinoblastoma protein (Rb) (67).

### p53 and senescence

Senescence is a form of cell cycle arrest that can be induced upon exposure to various stimuli either exposed endogenously or exogenously (76). The senescent cells can be characterized by their bigger size, a significant abundance of nucleoli and vacuoles compared to the normal cells (77). Cellular senescence could also be induced following the activation of p53 through several related target genes e.g., p21, p16-Rb, and BTG2 (67, 76, 78). Compared to the previously described p53-related mechanisms, apoptosis and cell cycle arrest, senescence also plays a pivotal role in preventing the progression of cancerous cells.

### Other p53 mechanisms in preventing cancerous events

In addition to these three main mechanisms of p53, other functions of p53 have also been deciphered. Those functions include p53 involvement in preventing cancer migration and metastasis, angiogenesis, cellular metabolism, oxidative stress, drug resistance, inducing autophagy, and promoting genomic stabilization (67, 79–84). Taken together, all of these mechanisms are crucial for preventing the genome from being cancerous.

### p53 mutations in gastrointestinal tract and associated cancers

As stated above, p53 mutations are found as the most mutated gene in many types of cancers, including cancers developed in the gastrointestinal tract, such as colorectal cancer (85, 86). p53 mutations can be detected in 34% of colon cancers occurring in the proximal area and in 45% of those occurring in the distal area of the colon (87). In addition to p53 genes, a number of other genes also experience mutations in colorectal cancer. However, a study conducted by Wood and co-workers indicated four other commonly mutated genes i.e., KRAS, APC, PIK3CA, and FBXW7 (86).

Furthermore, p53 mutations in colorectal cancer mainly occur in exon 5 to exon 8 in the DBD (p53 has 11 exons and 10 introns) (88). Throughout these vulnerable exons, several codons have been identified as the preferred sites of mutations (Table 3). For example, in exon 5, a missense mutation in codon 175 causes the failure to produce arginine. As a result, the codon is translated into histidine. This mutation occurs as codon CGC is changed into CAC. Another example is exhibited by missense alteration occurring in codon 282 in exon 8 where codon CGG (arginine) is changed into TGG (tryptophan) (68). Some other codons that are commonly vulnerable to mutations are codons 245, 248, and 273.

**Table 3:** Several common codons that are vulnerable to cellular stress leading to missense mutations (68).

**Exon (Codon)**	**Missense mutation**	**Amino acid change**
5 (175)	Guanine → Adenine	Arginine → Histidine
7 (245)	Guanine → Adenine	Glycine → Serine
	Guanine → Adenine	Glycine → Aspartic acid
7 (248)	Cytosine → Thymine	Arginine → Tryptophan
	Guanine → Adenine	Arginine → Glutamine
8 (273)	Cytosine → Thymine	Arginine → Cysteine
	Guanine → Adenine	Arginine → Histidine
8 (282)	Cytosine → Thymine	Arginine → Tryptophan

These data emerge other interesting questions. For example, why these exons and codons become the preferred sites of p53 mutations and why specific amino acid (e.g., arginine) seems to have higher vulnerability over the other amino acids (68). It has been demonstrated that p53 mutations are closely associated with several events leading to the malignancies. p53 mutations have a clear association with the emergence and development of cancer. Lopez et al. found that p53 mutations can be detected in more than 50% of colorectal cancer occurring sporadically (89). Not only, colorectal cancer, p53 mutations are also identified in high percentage in other types of gastrointestinal cancers. It is estimated that p53 mutations are involved in up to 77% of stomach cancer (90).

In addition, as in the other cancers, p53 mutations also show a close correlation with the progression and invasiveness of gastrointestinal cancer. One example is given by Russo et al. found that p53 mutations were closely linked to the invasiveness of colorectal cancer which is related to its ability to reach lymphatic and blood circulation providing a great chance to be metastatic to the other parts of the body (87).

### p53 limits the effectiveness of anticancer drugs

Most importantly, p53 mutation could diminish the effectiveness of chemotherapeutic agents (e.g., doxorubicin, temozolomide, tamoxifen, and gemcitabine) (80). Several mechanisms have been proposed to explain the emergence of this drug resistance (91, 92). p53 mutation could inhibit the entry of the chemotherapeutics so that the intracellular level of the drugs is inefficient in exerting their anticarcinogenic activities. Another proposed mechanism is related to the overexpression of ABC transporters, such as ABCB1 (p-glycoprotein) in cancerous cells, making the efflux of the drugs occurs extensively. The increased drug metabolism is also observed in p53 mutation which eventually causes extensive inactivation of the chemotherapeutics (91). Another study found that this effect was associated with the failure of the mutant p53 in upregulating PUMA expression (93). Although this study was only focused on colon cancer, this finding might correlate with the other types of cancer and provide a new insight for tackling mutant p53-related chemoresistance.

Molecularly, the involvement of the Wnt signaling pathway and epithelial-mesenchymal transition (EMT) in mediating pro-carcinogenic effects of the mutated p53 have attracted much interest. It has been found that proliferation, invasiveness, and development of colorectal cancer are facilitated by the upregulation of Wnt and EMT genes (94, 95). In the normal states, p53, in collaboration with microRNA-34, acts as a suppressor of the Wnt signaling pathway (94). A more recent study carried out *in vivo* investigating gastric cancer suggested that dysfunctionality of the wild-type p53 was accompanied by induction of Wnt and EMT. Also, this study found that loss of p53 function was negatively correlated with cyclooxygenase-2 (COX-2) levels indicating the role of inflammation in the progression of gastrointestinal cancer (96). In sum, the inhibition of these pathways (Wnt, EMT, and COX-2) might be useful in the effort of developing new chemotherapeutic agents.

### Drug candidates acting in p53 pathways

#### MDM2 inhibitors

Several drug candidates have been developed with promising potency in tackling the pro-carcinogenic effects of the mutated p53, especially in gastrointestinal cancers. The candidates working in inhibiting MDM2 activity may be the most attractive approach (97). As previously stated, MDM2 is the negative regulator of p53. It binds to the N-terminal of p53 initiating ubiquitination of the protein resulting in p53 degradation. It means that the excessive activity of MDM2 could lead to the significant inhibition of p53. As a result, this affects the tumor-suppressive function of p53. Conversely, inhibition of MDM2 could stabilize p53 protein (98).

Recently, several candidates with anti-MDM2 activities have been found and developed. Of those, MI-43 and nutlins are the most potent candidates. It has been reported that the former candidate has the ability to antagonize the action of MDM2 on p53. As a result, MI-43 could induce p53 accumulation leading to the activation of its target genes, such as p21, Noxa, and PUMA (99). A study reported that MI-43 could induce cell cycle arrest and apoptosis in colon cancer (100). Nutlins also have the ability to stabilize p53 by inhibiting MDM2 activity. Among the other Nutlins, Nutlin-3 activity in antagonizing MDM2 has been reported in the literature. In colorectal cancer, Nutlin-3 binds to the MDM2 pocket so that it disturbs the interaction between MDM2 and p53. As a result, p53 is stabilized and its downstream target genes are activated. However, it is noteworthy that Nutlin-3 has a great affinity only in MDM2, but not in the other members of the MDM family, such as MDMX. Consequently, cancer cells expressing abundant MDMX cannot be an appropriate target for Nutlin-3 (101).

Another MDM2 inhibitor with potential use in suppressing the growth of colon cancer is RITA (reactivation of p53 and induction of tumor cell apoptosis). Unlike the other MDM2 inhibitors that have been described above, this candidate binds directly to p53 instead of MDM2. This binding leads to the conformational change of p53 resulting in the interference with MDM2-p53 interaction (102).

#### Candidates targeting p63 and p73 genes

As stated previously, in addition to p53, the p53 family has two other proteins, p63 and p73. It has been demonstrated that targeting these members of the p53 family could be a promising strategy in developing anticancer drugs. For example, a derivative of ellipticine (a plant alkaloid), NSC176327, could kill the cancerous cells of colorectal cancer which is independent of p53 status. Also, this derivative could activate p73 and the target genes of the p53 family (e.g., p21 and DR5). Further, the loss of the p73 function plays a significant role in the emergence of chemoresistance (47).

#### Reactivator of mutant p53

Reactivation of mutant p53 is another strategy developed in the effort of seeking anticancer drugs. PRIMA-1 (p53-reactivation and induction of massive apoptosis-1) is an example of drugs utilizing this strategy (103). Reactivation of the mutated p53 by PRIMA-1 is linked to its covalent binding in DBD resulting in the restoration of a certain sequence in the core domain (104). Eventually, activation of p53 target genes in the cancer cells occurs. A more recent study reported the use of polysaccharides isolated from Ganoderma lucidum as an agent for restoration of the suppressive action of the mutated p53 in colorectal cancer (105).

## Relationship between compounds of cruciferous vegetables and p53 family in gastrointestinal tract and associated cancers

Many biological activities, such as DNA repair, cell cycle arrest, and apoptosis, depend on the tumor suppressor p53 (106). Approximately 50% of all malignancies in humans have p53 mutations (106). The majority of p53 mutations occur in the central core DNA-binding domain (DBD), which substantially impairs p53's ability to bind DNA and prevent tumor growth. Mutant p53 reduces the DNA-damage response and increases the resistance of tumor cells to drug-induced apoptosis (107). Additionally, there is evidence that suggests mutant p53 causes cancer by gaining function by transactivating genes related to growth or by silencing particular target genes (107). Increased tumorigenicity in nude mice and improved soft agar plating efficiency are both results of mutant p53 overexpression in deficient cells (107). In addition, p53 point mutation-carrying animals develop tumors and spread them more frequently than p53-deficient mice (108). Li-Fraumeni Syndrome, a germ-line p53 mutation, dramatically raises the chance of developing cancer in humans (108). Therefore, eliminating mutant p53 may present a viable strategy for cancer therapy and prevention. Cruciferous vegetables compounds have the capacity to arrest the cell cycle or induce apoptosis in cells by activating p53. For instance, carotene promotes the production of p53, p21, and BAX in cancer cells, which are all tumor suppressors (109). Activation of p53 and its targets p21, BAX, and RPRM led to the observation of the induction of cell cycle arrest when combined with bixin and canthaxanthin (110, 111). Apoptosis was seen in breast and bladder cancer cells after treatment with beta-carotene, phenethyl isothiocyanate, and allicin (112–114). The activation of BAX, Bcls, SAS, PERP, and LRDD resulted in this apoptosis, which was dependent on the protein p53. Natural compounds may be used to target these signaling pathways, ultimately halting the growth of cancer. For instance, astaxanthin, lycopene, and isothiocyanates, individually, have been found to induce p53-mediated apoptosis. The ERK, PI3K/Akt, and p21WAF1 pathways were activated in order to do this (115–117). Pharmacologically, therefore, MDM2 inhibition has become a promising mechanism utilized by a drug to exert its anticancer activity (118). Growth factors also play a crucial role in the development of cancer, including insulin-like growth factor (IGF), PDGF, EGF, tumor growth factor (TGF), FGF, and colony-stimulating factor (CSF). These substances stimulate signaling pathways, which in turn promote increased cell proliferation, block apoptosis, and allow cancer cells to invade healthy cells. Numerous downstream signaling pathways, including PI3K-AKT, Ras-MAPK, and others, are activated as a result of growth factor receptor activation (119). Additionally, it has been shown that isothiocyanates interact with the bladder epithelium and activate the cytoprotective enzymes GST and NQO1, which are known to detoxify carcinogens. Curiously, NQ01 has also been demonstrated to stabilize the p53 tumor suppressor. Additionally, it was shown that the bladder is one of the most receptive tissues to the activation of these enzymes by broccoli sprout extracts, suggesting that it may be particularly useful for guarding the bladder against the development of cancer (108). On the other side, angiogenesis makes cancer cells more resistant to the effects of chemotherapy and radiation while also playing a crucial role in the development, spread, and starts of cancer. In HUVEC, capillary tubes' ability to survive, migrate, and develop was diminished by both quercetin and benzyl isothiocyanate (120, 121). They also have success reducing the expression of angiogenic and metastatic markers. Additionally, it has been shown in a number of malignancies that methylation of the promoter regions of tumor-suppressor genes silences those genes (122). In addition, methylation of certain genes has been linked to resistance to radiation and chemotherapy (119). The suppression and reversal of the DNA methylation process may be one of the mechanisms by which natural substances exert their chemopreventive impact. In order to repair damaged DNA, it has been suggested that fucoxanthin and sulforaphane may inhibit and reverse DNA methylation, increase histone acetylation, and modify the structure of chromatin (123, 124). In summary, Crusiferous vegetable chemicals led to an increase in the tumor suppressor p53 protein level and transcriptional activity, which was accompanied by an increase in the levels of p21WAF1 and Bax, two of p53's transcriptional targets. Despite the fact that p21 is increased during p53-mediated G1 arrest, this occurrence does not lead to p53-induced apoptosis. The Bax/Bcl-2 ratio was altered by the p53-dependent increase in Bax expression, which also coincided with the activation of caspases 9 and 3 and PARP cleavage (109). Bax siRNA transfection of cells reversed these effects and prevented death, but it had no impact on the buildup of G1 cells. In conclusion, we suggest that p53-dependent pathways play a major role in cruciferous vegetable compound-mediated growth arrest and apoptosis. The area of the current investigation was to determine the function of p53 in cancer prevention and to clarify how it contributes to cell cycle arrest and apoptosis caused by Cruciferous vegetables compounds.

## Functional ingredients as therapeutics against gastrointestinal tract and associated cancers (pre-clinical findings)

Gastrointestinal (GIT) cancers, including those of the esophagus, stomach and colon, are closely associated with lifestyle factors, particularly diet (125). Dietary variables are thought to be responsible for one-third of all cancer deaths. A diet high in cruciferous vegetables like brussels sprouts and broccoli is linked to a lower risk of several cancers of gastro-intestinal tract (GIT). It has been found that cruciferous vegetables protect against GIT cancers more effectively than a diet rich in fruits and vegetables. p53 is a transcription factor that inhibits tumor growth and suppresses tumor growth. This protein regulates a wide range of physiological processes, including cell signaling, DNA damage response, genomic integrity, cell cycle regulation, and apoptosis. To stop cancerous or damaged cells from multiplying, p53 activates genes such as p21WAF1 and Bax, which in turn activate the apoptotic pathway. A lack of p21, a regulator of cell division, is caused by a failure of p53 to bind DNA, and lead to unchecked cell proliferation resulting in tumors (126). The tumor suppresses p53 during cancer cell growth (either silenced or mutated). In order to control cancer cell development, angiogenesis, and cancer cell migration, p53 must be expressed in cancer cells (127).

### Gastric cancer

**Figure 5:**
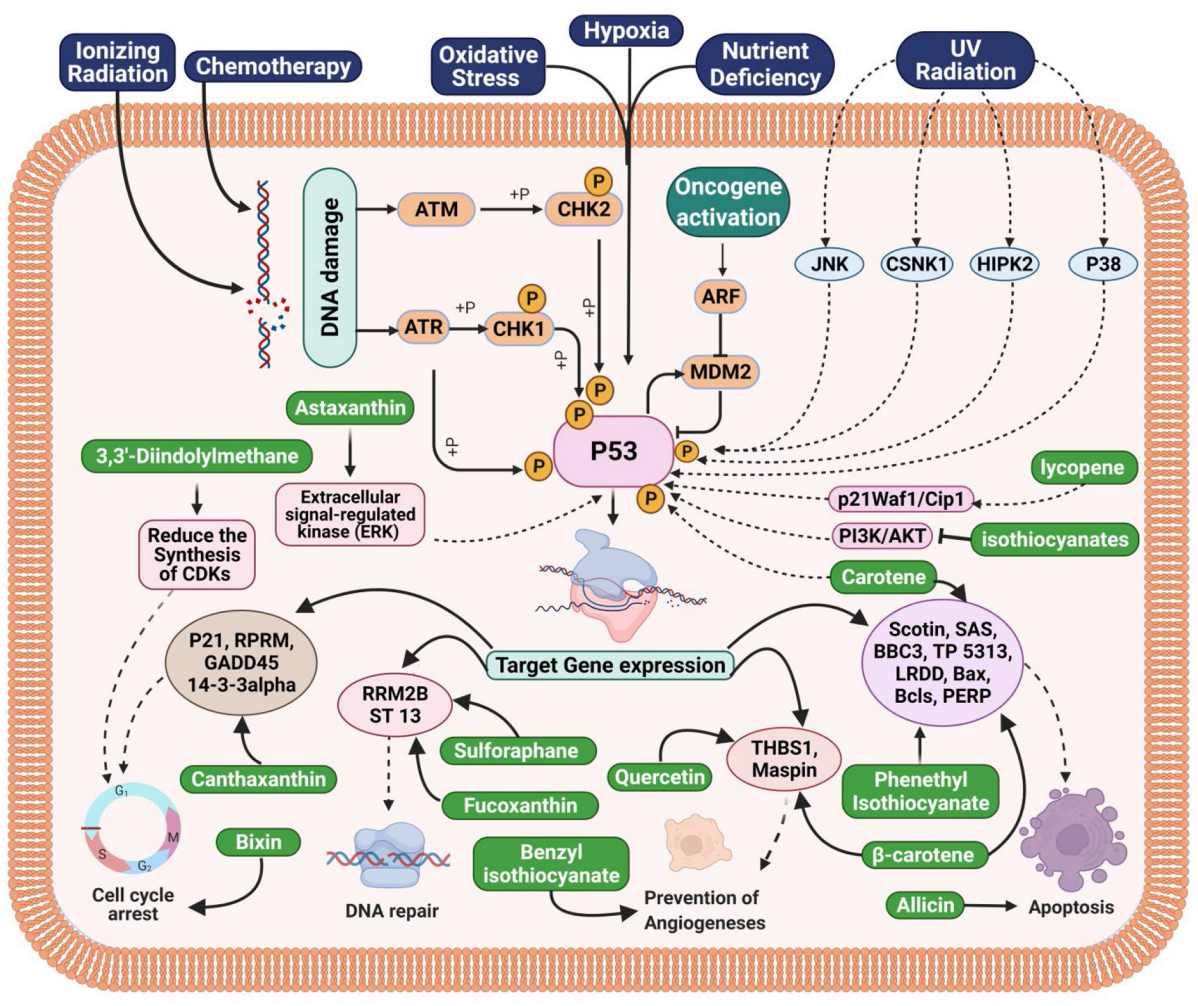
Functional foods bioactive compounds from cruciferous vegetables targeting p53 Family in gastrointestinal tract and associated cancers.

Isothiocyanates (ITCs), a hydrolysis product of glucosinolates present in cruciferous vegetables, are chemoprotective compounds synthesized by the enzyme myrosinase. Gastric cancer may benefit from the use of potential ITCs such as PEITC (phenethyl isothiocyanate), BITC (benzyl isothiocyanate), and SFN (sulforaphane) (128, 129). An anticancer possibility for gastric cancer has been examined using the nontoxic indole derivative 3,3'-Diindolylmethane (DIM) from cruciferous vegetables (130). It was observed that DIM inhibits gastric cancer growth *in vitro* and *in vivo*, depending on the dose, by Ye et al. (131). By stimulating the Hippo signaling system, DIM slowed the growth of gastric cancers in a xenograft mouse model, according to Li et al. Reduced synthesis of CDKs (cyclin-dependent kinases) 2, 4 and 6, as well as cyclin D1, allowed for G1 cell cycle arrest while increasing levels of p53 protein (132). Studies on laboratory animals have shown that carotenoids (lycopene, lutein, and β-carotene) prevent the formation and growth of chemotherapeutically produced gastric tumors (133, 134). There was an investigation into the effects of oral lycopene supplements in ferrets by Liu et al., who found that the p53 gene, its target genes (p21Waf1/Cip1 and Bax-1), and gastric mucosal cell proliferation and apoptosis were all affected by lycopene supplementation (135). Bax (Bcl-2 family member) and P21waf1/cip1 (CDK inhibitor) are both critical for apoptosis and G1 cell cycle arrest, respectively. p21waf1/cip1 and Bax-1 act together as mediators for the enhancement of p53-dependent necrosis (136). There was a 9-week experiment in which ferrets were exposed to cigarette smoke and given either modest or high dosages of lycopene supplementation. Ferrets given lycopene alone had significantly more lycopene in their gastrointestinal mucosa than ferrets given lycopene, and exposed to smoke, a finding that was dose-related (Figure 5). Lycopene dramatically reduced both the smoke-induced total p53 and phosphorylated p53 levels in ferrets that had recently been exposed to smoke, regardless of the level of total p53 and phosphorylated p53. In ferrets exposed to smoke alone, cell death indicators such p21 (Waf1/Cip1), Bax-1, and cleaved caspase 3 were significantly reduced, whereas lycopene dose-dependently mitigated the effects of smoke on these same markers as well as cyclin D1 and PCNA (Table 4) (135). Reactive oxygen species (ROS)-induced phosphorylation of p53 at serine 15 improves p53 accumulation and activation to prevent stomach cancer (159). It is possible to induce apoptosis in gastric cancer cells by activating p53 and upregulating the transcription of key target genes such as p21 (which is involved in cell cycle arrest) and pro-apoptotic BH3-only Bcl-2 family proteins, such as Bad, Noxa, BH3 interacting domain death agonist (Bid), and Bcl-2-like protein 11 (Bim; activator of BH3). Anti-apoptotic proteins like Bcl-2 and Bcl-2xL can be rendered inactive by the BH3 protein, a BH3-only sensitizer. There is a competition for the binding of BH3-only proteins like Bax and Bak, which results in their displacement from anti-apoptotic proteins like Bax and Bak (160).

**Table 4:** Functional ingredients from cruciferous vegetables in gastrointestinal tract and associated cancers.

**Gastrointestinal cancer types**	**Functional ingredients**	**Study model**	**Doses**	**Findings**	**References**
Gastric cancer	Curcumin	*In vitro* (SGC7901, BGC823, MGC803 and MKN1 cell line)	50–100 μM	Efficient chemo sensitizing effect and also inhibits viability, proliferation, and migration of gastric cancer cells mainly	(137)
	Quercetin	Human model	3.89–6.02 mg/day	Inhibited cell growth and induced apoptosis, necrosis, and autophagy	(121)
	Allicin	*In vitro* (SGC-7901 cancer cells)	15–120 μg/ml	Apoptotic activity	(112)
	β-carotene	Human cell line	0–6.2 μg/dl	Reduced risk of gastric cancer	(113)
	Isothiocyanate	Human model	0.1 μmol/L.	Effective in protecting against gastric cancer, particularly among those who were lack of genes GSTMI (glutathione S-transferase M1) and GSTTI (glutathione S-transferase T1)	(138)
	Sulforaphane	Gastric cancer stem cells (CSCs)	0, 1, 5, 10 μM	Inhibitory action of sulforaphane on gastric CSCs *via* suppressing Sonic Hh pathway	(139)
	Thioredoxin reductase (TR)	Human model	7.34 U/mL	Threshold of TrxR activity was distinctive in the diagnosis of different tumor types	(140)
	Astaxanthin	Human gastric adenocarcinoma cell lines (AGS, KATO-III, MKN-45, and SNU-1)	0, 10, 50, and 100 μM	Astaxanthin inhibits proliferation by interrupting cell cycle progression in KATO-III and SNU-1 gastric cancer cells	(117)
	Benzyl isothiocyanate	AGS human gastric cancer cells	0, 0.25 and 0.5 mM	Inhibit migration and invasion of human gastric cancer AGS cells	(120)
Small Intestine cancer	Phenyl isothiocyanate	Human model	0.2–25 mmol/L	Isothiocyanate exposure may reduce the risk of colorectal cancer	(114)
	Thioredoxin reductase (TR)	Human model	-	Controls cell development by providing the reducing power for p53 and the redox cycling of endogenous antioxidants	(141)
	Sulforaphane	GC cell lines	0–22.5 μM	Role in p53 stabilization and nuclear localization	(142)
	Astaxanthin	Small intestine carcinoma cell lines	0, 10, 50, and 100 μM	Interrupting cell cycle progression	(117)
	Curcumin	Mice model	1,000 mg/kg	Suppressed Nrf2-Dependent Genes in Small Intestine	(143)
	Quercetin	Mice model	2% in diet	Anti-tumor activity in the small intestine	(144)
	Benzyl isothiocyanate (BITC)	Rat Model	400 P.P.M	Promising chemopreventive agents for human intestinal neoplasia	(145)
	Sulforaphane	Mice model	300 and 600 p.p.m.	Developed significantly less and smaller polyps with higher apoptotic and lower proliferative indices in their small intestine	(146)
Colon cancer	Isothiocyanate	Colon cancer cell lines	2.5 mM	Block the (PI3K)/AKT-dependent survival pathway of colon cancer cell lines, while stimulating the p53 pathway	(116)
	BITC	HCT-116 cells	50 μM	Capable of ameliorating the inflammation associated with colon cancer	(116)
	Sulforaphane	HCT116 colon cancer cells		DNA repair protein causes DNA damage in colon cancer cells	(123)
	PEITC	HT29 colon cancer cells	10–50 μM	Have anti-metastatic and anti-inflammatory effects against colon cancer	(147)
	3,3′-diindolylmethane (DIM)	Colon cancer HT29 cells	100 μM	Cytotoxic effects	(148)
	β-carotene	Human models	-	Increase Bax and P53 levels in malignant colon cells while decreasing Bcl-2 levels	(149)
	Astaxanthin	HCT-116 colon cancer cells	5–25 μg/ml	Increase of p53, p21WAF-1/CIP-1 and p27 expression (220, 160, 250%, respectively) was observed, concomitantly with a decrease of cyclin D1 expression (58%) and AKT phosphorylation (21%).	(150)
	Bixin	CRC cell lines	0-80 μM	Inhibit the CRC cell proliferation and survival	(111)
	β-cryptoxanthin	Human models	-	Enhances the antitumoral activity of oxaliplatin through δnp73 negative regulation in colon cancer	(151)
	Lycopene	Colon cancer HT-29 cells	2, 5, 10 μM	Inhibited cell proliferation in human colon cancer HT-29 cells	(115)
Hepatic and Pancreatic cancer	Bixin	Hep3B cell	5-50 μg/ml	Cell growth inhibition	(110)
	Quercetin	PANC-1	0, 10, 25, 50, 100, or 200 μM	Shows significant pro-apoptotic effects	(152)
	Curcumin	Hepatic cancer human models	-	Inhibited MMP-9 secretion in HCC (CBO140C12) cells, and repressed the adhesion and migration of fibronectin and laminin	(153)
	β-cryptoxanthin	Human models	-	Decreased significantly with increased prevalence of Leiden mutation (as a genetic factor) in patients before the clinical manifestation of histologically different GI cancer	(154)
	Lycopene	Hep3B human hepatoma c	-	Induced G0/G1 arrest and S phase block and inhibited cell growth in a dose-dependent manner by almost 40%	(155)
	Astaxanthin	HepG2 hepatoma cells	25 and 42 μM	Arrest induction at G0/G1 phase	(156)
	Fucoxanthin	Mice models	488.8 mg Fx/kg bw	Mediates the suppression of the CCL21/CCR7 axis, BTLA, tumor microenvironment, epithelial mesenchymal transition, and adhesion	(124)
	Isothiocyanate sulforaphane	MIA PaCa-2 and PANC-1.	10 μmol/L sulforaphane.	Sulforaphane Suppressed Growth and Triggered Activation of Caspase-3- and Caspase-8-Dependent Cell Death	(157)
	BITC	Mice model	0.5 μmol/L in plasma	BITC-treated mice showed 43% less tumor growth	(158)

### Small intestine cancer

The presence of ITCs, particularly BITC, has been linked to a lower risk of small intestine cancers or cancer consequences in people who eat cruciferous vegetables. Cruciferous vegetables may contain ITCs that may be useful in the treatment of small intestinal malignancies, including BITC, PEITC, and SFN (128, 129). Thioredoxin reductase (TR), a selenoprotein that lowers thioredoxin and controls cell development by providing the reducing power for p53 and the redox cycling of endogenous antioxidants including vitamin C, lipoic acid, and niacin, can be made more active by ITCs and carotenoids present in cruciferous vegetables (Figure 4) (141). Apoptosis of cancer cells can be induced by increasing the expression and stability of the cell-killing protein p53, as well as PEITC and sulforaphane's role in p53 stabilization and nuclear localization (141, 142). Jang et al. and Shin et al. reported that carotene can promote apoptosis in intestinal cellular epithelium by boosting p53 and lowering Bcl-2, which is anti-apoptotic (Figure 5) (133, 161). Kim et al. conducted an *in vitro* investigation to evaluate the mechanism of astaxanthin's anticancer effects on small intestine carcinoma cell lines. Astaxanthin slowed down the growth of small intestinal cancer cells. The phosphorylation of extracellular signal-regulated kinase (ERK) was suppressed, and the expression of p53 was shown to rise as a result (Table 4) (117).

### Colon cancer

Oncogenes including K-ras and adenomatous polyposis coli (APC), as well as tumor suppressor genes like Smad4 and p53, have a critical role in colon cancer formation (162, 163). PI3K/AKT/mTOR pathway and p53 pathway abnormalities are the most prevalent anomalies in most colon cancer cells (164, 165). Blocking the phosphatidylinositol-3-kinases (PI3K/AKT) pathways while activating the p53 pathway; slows the growth of cancer cells. These pathways affect glucose metabolism, apoptosis, cell proliferation, and migration (166). ITCs present in cruciferous vegetables such as BITC, PEITC and sulforaphane have antimetastatic properties against colon cancer. These ITCs block the (PI3K)/AKT-dependent survival pathway of colon cancer cell lines, while stimulating the p53 pathway. In addition, they increase apoptosis-related proteins due to activation of p53-family genes, while decreasing metastasis-related proteins. Because of all these reasons, BITC, PEITC and sulforaphane were capable of ameliorating the inflammation associated with colon cancer (116, 167). In HT29 colon cancer cells, BITC and PEITC have been demonstrated to have anti-metastatic and anti-inflammatory effects against colon cancer, and it slowed the migration of colon cancer cells through the activation of p53 pathway (147, 168, 169). Sulforaphane (SFN)-induced acetylation of a DNA repair protein causes DNA damage in colon cancer cells (Figure 5) (123). The p53-stimulated apoptosis of colon cancer cells is induced by activation of detoxifying enzymes, the release of cytochrome c, and the stimulation of poly(ADP-ribose) polymerase (PARP) proteolysis (170–172). Apoptosis occurs as a result of cell cycle arrest (173). Using human colorectal cancer cells as a model, Myzak et al., found that sulforaphane decreased histone deacetylase (HDAC) activity while simultaneously increasing histone acetylation (174). HDAC inhibition has been linked to an increase in the transcription of p53 gene, which is a tumor-suppressor gene. Lycopene and canthaxanthin are two carotenoids that inhibited colon carcinoma cell proliferation by altering the cell cycle (175). As a result of cell cycle arrest due to activation of p53 pathway, astaxanthin was demonstrated to reduce cell proliferation and trigger death in cancer cells (Table 4) (117). The study by Palozza et al., found that *Haematococcus pluvialis* reduced cyclin D1 levels in colon cancer cells while increasing p53 and p21WAF-1/CIP1 levels in the cells (117). As a result of these findings, it can be concluded that the G1-phase cell cycle is blocked. In addition, β-carotene extracted from cruciferous vegetable has been demonstrated to increase Bax and P53 levels in malignant colon cells while decreasing Bcl-2 levels (149, 176).

### Hepatic and pancreatic cancer

Anti-apoptotic genes overexpressed and tumor suppressor genes mutated, such as p53, are the first signs of tumor formation in the liver and pancreatic (158, 177). Several carotenoids in cruciferous vegetables, including bixin, β-cryptoxanthin, lutein, lycopene, astaxanthin, and fucoxanthin, have been examined for their function in triggering apoptosis in hepatic and pancreatic cancer cells through ROS generation. When the anti-apoptotic Bcl-2 and xL proteins, as well as proteins associated with B-cell lymphoma 2 (Bcl-2), are inhibited and pro-apoptotic proteins like p21, p27, and p53 are activated, hepato-pancreatic cancer cells might undergo apoptosis (149, 178). Dietary lutein raised the mRNA expression of proapoptotic genes p53 and Bax, decreased the expression of antiapoptotic gene Bcl-2, and increased the Bax:Bcl-2 ratio in hepato-pancreatic cancers (Figure 5). Activation of the p53 tumor suppressor gene causes apoptosis, DNA damage tolerance, and DNA repair (Table 4) (179). Further studies have demonstrated that the cruciferous vegetable isothiocyanates stimulate the GST and NQO1 enzymes renowned for their ability to remove carcinogens from the cells of the hepatic and pancreatic epithelial tissue. NQ01 appears to stabilize the tumor suppressor p53, according to certain research results. Liver and pancreas are the tissues susceptible to the stimulation of these enzymes by broccoli sprout extracts, which may be particularly effective in protecting these organs against the onset of cancer (119, 180).

## Current progress toward clinical applications

The ability of cruciferous bioactive chemicals to suppress cancers in experimental animals has been documented in numerous studies. At the same time, the research studies conducted on human beings are very less. Isothiocyanates, indole-3-carbinol, and phytoalexins are among the bioactive chemicals in cruciferous vegetables studied for prevention of cancer of gastro-intestinal tract and other associated cancers (181). Cell cycle, apoptosis, genomic integrity, and DNA repair in human beings are all controlled by the tumor suppressor gene p53. Angiogenesis inhibition, cell cycle inhibition, and genetic stability come from the activation of particular genes by activated p53 gene (19). In addition, active p53 can operate as both a transactivator and a transrepressor at the same time. Activating p53 by the bioactive chemicals present in cruciferous vegetables may cause cell cycle arrest or death in cancerous cells of gastro-intestinal tract (129, 182). Carotenoids, for example, activates p53 and its targets p21 and Bax in gastric and colon cancer cells (183). Cell cycle arrest and apoptosis were both induced by lutein, which also activated the p53 gene's targets, including p21, Bax and PUMA (p53-upregulated modulator of apoptosis). Using ITCs, human gastric and colon cancer cells were able to apoptosis through the p53-dependent BAX induction. Apoptosis and ROS (reactive oxygen species) generation are two ways that ITCs limit tumor growth in body tissues of human beings. As ITCs and β-carotene activates p53's mitochondrial translocation, it has been shown to incite cell death through p53-mediated mechanisms. There are two closely related proteins in mammalian cells that when stimulated can cause apoptosis, similar to p53 protein, which are known as p63 and p73 (149). Indole-3-carbinol and phytoalexins have been found to induce apoptosis and cell cycle arrest in HT-29 human colon cancer cells (8). DNA damage checkpoints are essential for genome integrity because they stop the progression of the cell cycle when DNA damage or incomplete replication occurs, and the arrest of the G1 checkpoint occurs in mammalian cells because p53 protein mediates the action (182). Li et al. investigated the effects of indole-3-carbinol extracted from cruciferous vegetables on p53 protein induction. Indole-3-carbinol therapy enhanced the p53 protein level in human gastric cancer cell lines, causing cell cycle arrest in the G1 phase (132). By modifying the amounts of proteins that control cell cycle progression, indole-3-carbinol prevented human gastric cancer cells from entering the G1 phase. Glucosinolate compounds present in seeds of cruciferous vegetables, such as sinigrin, have been demonstrated to dramatically suppress hepatotumor cell proliferation when administered through the p53 pathway. Sinigrin, a major glucosinolate in cruciferous vegetable seeds, has been shown to suppress the growth of human liver cancer cells in a way that is dependent on the p53 signaling pathway (184, 185). More research studies should be conducted in the near future to attain further more development with regard to clinical applications of bioactive compounds available from cruciferous vegetables in human beings.

## Concluding remarks and futuristic vision

A synopsis of the cancer-preventive potential of numerous Brassicaceae family members has been reported in this review. Even though there is a strong correlation between the prevention of carcinogenesis and consumption of cruciferous vegetables, it must be emphasized that many more studies are needed to fully understand the influence of these functional foods bioactive compounds on the human body. The upcoming experiments must specifically address the questions of bioavailability, stability, transport, and metabolism. These substances may have synergistic effects. It needs to be confirmed in further studies. In terms of the cancer-preventive characteristics of phytochemicals included in these veggies, the additional effects of typical food preparation techniques constitute another component that has yet to be fully investigated. Numerous studies aided in the acceptance of dietary agents as cancer treatment options. Due to their anti-tumorigenic and anti-proliferative competences, cruciferous vegetables are abundant with many functional bioactive compounds that have noteworthy inhibitory effects on different pathways of cancer cells. These veggies are advantageous because they are precursors to glucosinolates, which are precursors to isothiocyanates like sulforaphane and indoles like indole-3-carbinol. HDAC and DNMT overexpression, as well as miRNA misexpression, are common features of most malignancies. I3C, SFN, and I3C are regulators and inhibitors of these processes, and their usage causes malignant cell lines to seem more normal and healthier. With the inclusion of SFN and I3C, an increase in programmed cell death, as well as substantial reductions in uncontrolled cell proliferation, have been observed. Studies showed that functional bioactive compounds from cruciferous vegetables are potential candidates to fight cancer. Future research will likely focus on determining the epigenetic events influenced by the bioactive components of cruciferous vegetables and their importance in not just cancer prevention but also a variety of other biological systems.

## Author contributions

SM, DC, and TE conceptualized and designed the manuscript, participating in drafting the article and/or acquisition of data, and/or analysis and interpretation of data. SM, BZ, RD, SSM, AM, MS, and FN participating in drafting the article and prepared the figures and tables. SM, TE, MK, AI, and JS-G wrote, edited, and revised the manuscript critically. TE and JS-G revised the final written. All authors critically revised the manuscript concerning intellectual content and approved the final manuscript.

## Funding

The authors express their gratitude to Research Center of Advanced Materials, King Khalid University, Saudi Arabia, for support (award number KKU/RCAMS/22). Funding for open access charge: Universidade de Vigo/CISUG.

## Conflict of interest

The authors declare that the research was conducted in the absence of any commercial or financial relationships that could be construed as a potential conflict of interest.

## Publisher's note

All claims expressed in this article are solely those of the authors and do not necessarily represent those of their affiliated organizations, or those of the publisher, the editors and the reviewers. Any product that may be evaluated in this article, or claim that may be made by its manufacturer, is not guaranteed or endorsed by the publisher.
